# Deep Eutectic Solvents as Phase Change Materials in Solar Thermal Power Plants: Energy and Exergy Analyses

**DOI:** 10.3390/molecules27041427

**Published:** 2022-02-20

**Authors:** Hamed Peyrovedin, Reza Haghbakhsh, Ana Rita C. Duarte, Alireza Shariati

**Affiliations:** 1 School of Chemical and Petroleum Engineering, Shiraz University, Shiraz 71345-51154, Iran; hamed.p.2012@gmail.com; 2 Department of Chemical Engineering, Faculty of Engineering, University of Isfahan, Isfahan 81746-73441, Iran; r.haghbakhsh@eng.ui.ac.ir; 3 LAQV, REQUIMTE, Departamento de Química da Faculdade de Ciências e Tecnologia, Universidade Nova de Lisboa, 2829-516 Caparica, Portugal; ard08968@fct.unl.pt

**Keywords:** DES, green solvent, solar energy, Rankine cycle, PCM, exergy analysis, energy analysis

## Abstract

Nowadays, producing energy from solar thermal power plants based on organic Rankine cycles coupled with phase change material has attracted the attention of researchers. Obviously, in such solar plants, the physical properties of the utilized phase change material (PCM) play important roles in the amounts of generated power and the efficiencies of the plant. Therefore, to choose the best PCM, various factors must be taken into account. In addition, considering the physical properties of the candidate PCM, the issue of environmental sustainability should also be considered when making the selection. Deep eutectic solvents (DESs) are novel green solvents, which, in addition to having various favorable characteristics, are environmentally sustainable. Accordingly, in this work, the feasibility of using seven different deep eutectic solvents as the PCMs of solar thermal power plants with organic Rankine cycles was investigated. By applying exergy and energy analyses, the performances of each were compared to paraffin, which is a conventional PCM. According to the achieved results, most of the investigated “DES cycles” produce more power than the conventional cycle using paraffin as its PCM. Furthermore, lower amounts of the PCM are required when paraffin is replaced by a DES at the same operational conditions.

## 1. Introduction

Power generation using fossil fuels is the most commonly used method throughout the world. One of the most significant disadvantages of using fossil fuels is the release of greenhouse gases, such as carbon dioxide, into the atmosphere [1,2,3]. Accordingly, various sustainable methods, such as the use of low-grade heat [4,5], geothermal energy [6,7], wind energy [8,9], and solar energy [10,11,12,13,14], have been applied to produce clean energy with little environmental pollution. Among these novel methods, harnessing solar energy via solar thermal power plants coupled with the Rankine cycle has gained attention [10,11,12,13,14]. In such plants, the collected solar energy is transformed to heat, being used by the Rankine cycle to generate power by use of a turbine [10,11,12,13,14]. However, the greatest disadvantage of solar energy plants is the limited availability of solar radiation on cloudy days and, also, at night. In order to overcome this issue, the utilization of thermal energy storage (TES) systems incorporating phase change materials (PCMs) was introduced to achieve uniform power generation [15,16]. Actually, PCMs consist of various groups of materials with high heat capacities, capable of storing and releasing energy using their latent and sensible heats [17]. In recent years, the potentials of different materials, such as organic and inorganic materials, were studied as PCMs in a variety of processes, including heating and cooling processes, solar energy storage, and the food industries [17,18,19,20]. In solar thermal power generation plants, different types of materials were considered as PCMs, including both organic and inorganic material and conventional eutectic mixtures [21]. However, all of the aforementioned materials have certain shortcomings. For instance, organic PCMs, such as paraffin, are flammable and their volume changes are relatively large. Inorganic PCMs, such as metallic PCMs, are mostly corrosive and have issues of high-volume change upon temperature changes. Regarding conventional eutectic mixtures, they have very high melting-point temperatures, and so, are limited to only certain high-temperature applications [21,22]. Moreover, most of the thermodynamic properties of eutectic PCMs are unknown [21].

According to the required properties for each process, various materials are available to consider as PCMs, however, nowadays, it is more vital than ever to consider only those that are environmentally friendly. One such category of sustainable material, having only recently been introduced to the research community by Abbott et al. in 2003 [23], is the Deep Eutectic Solvent (DES). These sustainable solvents also have the potential to be applied as PCMs [21]. A DES is actually a mixture of two or more components, including one hydrogen bond acceptor (HBA) and one or more hydrogen bond donors (HBD). DESs have many advantages such as low vapor pressure, biodegradability, sustainability, non-flammability, ease of preparation, and low cost. Furthermore, they are mostly nontoxic [4,5,24,25]. In addition, the most unique characteristic of DESs is the ability to tune their physical properties. Since combinations of numerous HBA and HBD components are possible, as well as various ratios of the two, countless types of DESs with different physical properties can be envisioned. Therefore, by setting the required physical properties for each specific application, the most favorable DES can be specifically engineered for the purpose. Due to the multitude of advantages, the applications of DESs in various processes are being investigated, including, for example, extraction [25,26], electrochemistry [25,27], absorption [4,5], and chemical reactions [25,28]. However, studies investigating the feasibility of using DESs as PCMs in solar thermal power plants are quite rare [26].

The only published study in open literature considering DESs as PCMs is that of Shahbaz et al., which considered the application of a calcium chloride hexahydrate-based DES as a PCM for thermal-comfort building applications. They prepared five DESs using choline chloride and CaCl_2_.6H_2_O with different HBA to HBD molar ratios and reported their thermal properties. According to their thermal cycling tests, they claimed that the two DESs of choline chloride: CaCl_2_.6H_2_O with the molar ratios of 1:6 and 1:8, can potentially be used for the thermal comfort processes in buildings. However, they did not consider energy and exergy analyses for their suggested process [26].

Based on the very favorable characteristics of DESs, and the benefits of replacing conventional PCMs with environmentally sustainable material in solar thermal power generation plants, the feasibility of using various DESs as PCMs in solar thermal power generation cycles was investigated in this study. For this purpose, a conventional solar thermal power generation cycle was modified, and then, by employing energy and exergy analyses, the performances of all the cycles considering seven different DESs as PCMs were studied.

## 2. Method

### 2.1. The Modified Solar Thermal Power Generation Cycle

**Figure 1:**
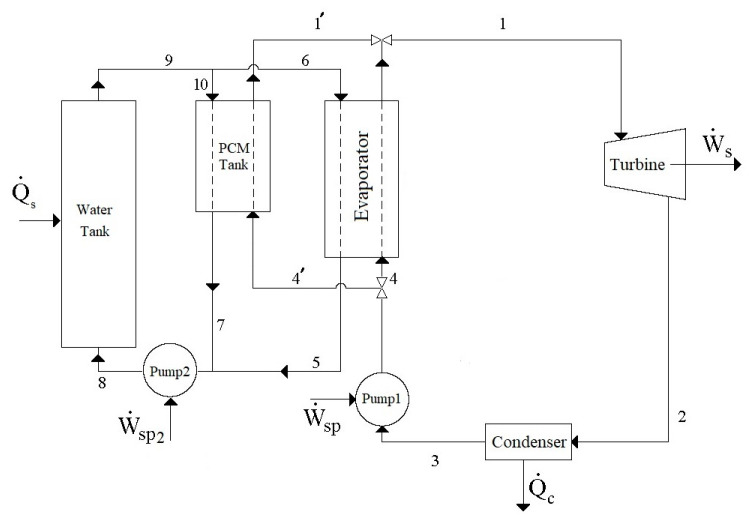
Schematic diagram of the modified solar thermal power generation cycle.

The schematic diagram of the modified cycle under consideration is presented in Figure 1. According to this cycle, for 12 hours during the day, the heating fluid (liquid water) enters the water tank as Stream 8, which receives solar energy that is collected by collectors as heat $\dot{Q_{s}}$ and leaves the water tank as Stream 9. The heated liquid water in stream 9 is separated into the two streams of 10 and 6. Stream 10 enters the PCM tank, which contains a DES as the PCM for absorbing heat from entering the heated water (Stream 10) during the day. The cooled liquid water then leaves the PCM tank as Stream 7. In this mode, the PCM tank is in the “charging” state to increase its energy. The other heated water stream (Stream 6) enters the evaporator and provides the required heat for the working fluid (R134a) of the Rankine cycle during the day and leaves the evaporator with lower energy as Stream 5. This leaving stream is finally combined with Stream 7 and the resulting stream is recycled to the water tank for continuing the cycle. On the other hand, in the evaporator of the Rankine cycle, the working fluid (R134a) in Stream 4 absorbs heat from the heated water and is evaporated. Evaporated R134a, with high pressure and temperature, enters the turbine as Stream 1 and produces power, $\dot{W_{s}}$. Following power production, the low-temperature–low-pressure vapor of R134a enters the condenser as Stream 2 and desorbs heat, $\dot{Q_{c}}$, to become liquified and leave the condenser as Stream 3. The pressure of liquified R134a is increased using Pump 1 and the pressurized R134a is recycled to the evaporator as Stream 4 for receiving heat once more from the heated water and continuing the Rankine cycle. However, during the night (for 12 h), the required heat for evaporating R134a in the evaporator is provided by the PCM tank which is now in the energy discharging mode. Accordingly, during the night, the liquified R134a (Stream 4) enters the PCM tank as Stream 4′ instead of entering the evaporator as Stream 4. In the PCM tank, the liquified R134a absorbs heat, $Q_{PCM,night}$, and upon evaporation, it enters the turbine as Stream 1′. Accordingly, during the night, Streams 5–10 which are responsible for transferring solar energy to R134a in the Rankine cycle by the water tank are shut off, and so, the required energy of the Rankine cycle is provided only by the charged PCM tank. By this design, the power production process continuously operates, both day and night, at a constant rate.

### 2.2. Energy Analysis

The energy analysis of the modified solar thermal power generation cycle is applied by considering the energy balance for all of the units of the investigated cycle. For the energy analysis, a number of usual assumptions considered in literature studies [4,5,12,29,30], are also considered here.

- The pressure drops (i.e., the required shaft work for Pump 2) in the pipes, PCM tank, condenser, and evaporator are neglected. Additionally, heat losses of the pipelines are neglected;
- Stream 3 is considered as saturated liquid R134a at the condenser pressure;
- The turbine’s isentropic efficiency is considered to be equal to 0.75;
- The PCM tank is well insulated;
- The required work of Pump 1 is negligible in comparison to the produced work of the turbine;
- The outlet water from the water tank (Streams 6, 9, and 10) is saturated liquid;
- The mass flow rate of the outlet water from the water tank (Stream 9) is split equally into Streams 6 and 10. Then, mass flow rates of the water entering the PCM tank and evaporator are the same during the day;
- The general cycle properties, excluding the received solar energy, remains constant during day and night;
- Day and night hours are considered equal, as 12 h.

According to these common assumptions, by applying the first law of thermodynamics to all of the equipment of the investigated cycle, energy analysis is considered.

Equations (1)–(3) show the applied energy balances for the turbine, condenser, and evaporator, respectively.

(1) $$\left|{\dot{W}}_{s}|={\dot{m}}_{r}(h_{1}-h_{2})={\dot{m}}_{r}(h_{1^{\prime }}-h_{2}\right)$$

where $h_{1}$ and $h_{2}$ are the specific enthalpies of the inlet and outlet streams of the turbine, respectively. Additionally, $h_{1}$ and $h_{1^{\prime }}$ are considered for the day and night, respectively. $\dot{m_{r}}$ and $\dot{W_{s}}$ are the mass flow rate of the working fluid and the produced power of the Rankine cycle, respectively. 

For the condenser,

(2) $$\left|\dot{Q_{c}}|=\dot{m_{r}}(h_{2}-h_{3}\right)$$

where $h_{2}$ and *h_3_* are the specific enthalpies of the inlet and outlet streams of the condenser, respectively. $\dot{Q_{c}}$ is the desorbed heat from the working fluid of the Rankine cycle.

For the evaporator, which is used only during the day,

(3) $$\dot{m_{w}}(h_{6}-h_{5})=\dot{m_{r}}(h_{1}-h_{4})$$

where $\dot{m_{w}}$ represents the mass flow rate of the heating fluid (water), and *h*_6_ and *h*_5_ are the specific enthalpies of the inlet and outlet heating working fluid streams (water) of the evaporator, respectively. *h*_4_ and *h*_1_ are the specific enthalpies of the inlet and outlet Rankine cycle working fluid streams (R134a) of the evaporator, respectively. 

For the PCM tank, the energy balance is investigated separately for day and night.

- *During the Day*
  The PCM tank is charged during the day by absorbing heat from the heating fluid (water). Accordingly, the energy balance of the PCM tank during the day follows Equation (4).
  (4) $$Q_{PCM,day}=m_{PCM}\Delta h_{fus,PCM}=t_{charging}\dot{m_{w}}(h_{10}-h_{7})$$
  where $m_{PCM}$ is the mass of the PCM, $\Delta h_{fus,PCM}$ is the PCM enthalpy of fusion, and $t_{charging}$ is the charging time in the day, equal to 12 h. $Q_{PCM,day}$ is the heat absorbed by the PCM from the heating fluid (water) during the day. Streams $4^{\prime }$ and $1^{\prime }$ are shut down during the day and the only inlet and outlet streams of the PCM tank are Streams 10 and 7, whose specific enthalpies are shown as $h_{10}$ and $h_{7}$.
- *During the Night*
  The PCM tank is discharged during the night by desorbing heat to the Rankine cycle working fluid (R134a). Therefore, the energy balance of the PCM tank during the night follows Equation (5).
  (5) $$\left|Q_{PCM,night}|=m_{PCM}\Delta h_{fus,PCM}=t_{discharging}\dot{m_{r}}|h_{1^{\prime }}-h_{4^{\prime }}\right|$$
  where $t_{discharging}$ is the discharging time during the night, equal to 12 h. $Q_{PCM,night}$ is the desorbed heat by the PCM to the Rankine cycle working fluid (R134a) during the night. Streams 10 and 7 are shut down at night, therefore, the only inlet and outlet streams of the PCM tank are Streams $4^{\prime }$ and $1^{\prime }$, with specific enthalpies of $h_{4^{\prime }}$ and $h_{1^{\prime }}$, respectively.

For the water tank, which is used only during the day, the energy balance is,

(6) $$4^{\prime }m_{w}C_{p_{w}}\frac{dT_{w}}{dt}=\dot{Q_{s}}+{\dot{m}}_{w_{9}}(h_{8}-h_{9})$$

where $h_{8}$ and $h_{9}$ are the specific enthalpies of the inlet and outlet streams of the water tank, respectively. $\dot{Q_{s}}$ is the collected solar energy. ${\dot{m}}_{w_{9}}$ is the mass flow rate of Stream 9 and based on the proposed assumptions, it is twice the mass flow rate of Streams 10 (or 6). Therefore,

(7) $${\dot{m}}_{w_{9}}=2{\dot{m}}_{w}$$

In Equations (6) and (7), ${\dot{m}}_{w}$ and $T_{w}$ are the total mass and the temperature of water in the water tank, respectively, and $C_{p_{w}}$ is the heat capacity of water. In this study, it is assumed that the collected solar energy is controlled carefully using controlling collectors, therefore, the water tank during the day is at a thermal steady state. In this way, the unsteady state term of Equation (6) can be neglected. This assumption is, in fact, easily obtainable because during the day, the amount of collected solar energy which is transferred to the water tank is controlled in a way to keep the water at its boiling point, and since a pure component boils at a constant temperature, the temperature of water in the water tank remains constant. In this way, there is no temperature change in the water tank. So, during the day, Equation (6) can be simplified as follows.

(8) $$\dot{Q_{s}}={\dot{m}}_{w_{9}}(h_{9}-h_{8})$$

### 2.3. Exergy Analysis

Exergy analysis is a way to define how far a system operates from ideal conditions. Exergy indicates the maximum amount of work that a system can generate under the second law of thermodynamics. Consequently, since all real systems are far from their ideal state, they cannot produce the maximum theoretical amount of work, and some of the theoretical maximum is wasted as exergy destruction [31].

For a steady-state process, the destruction of exergy for equipment *i* (${\dot{E}}_{d,i}$) is generally determined based on Equations (9) and (10) [32,33].

(9) $${\dot{E}}_{{}_{d,i}}=\sum_{j}{\left(\dot{m_{j}}e_{j}\right)}_{in}-\sum_{k}{\left(\dot{m_{k}}e_{k}\right)}_{out}+\sum_{}\dot{W_{in}}-\sum_{}\dot{W_{out}}+\sum_{}\dot{[Q}(1-\frac{T_{0}}{T}){]}_{in}-\sum_{}\dot{[Q}(1-\frac{T_{0}}{T}){]}_{out}$$

(10) $$e_{i}=(h_{i}-h_{0})-T_{0}(s_{i}-s_{0})$$

In Equation (9), the first and second terms of the right-hand side show the input and output exergies by the streams for equipment *i*. The third and fourth terms show the exergy changes of equipment *i* owing to the work transferred, and, finally, the last two terms of the right-hand side of Equation (9), represent the exergy changes due to the heat transferred [32,33]. In this equation, $T_{0}$ is the surrounding temperature, considered as 273.15 K, which is also the selected reference temperature. *T* is the temperature of the equipment. In Equation (10), $h_{0}$ and $s_{0}$ are the enthalpy and entropy of the environment, considered at the reference conditions (i.e., at the reference temperature of $T_{0}$ and reference pressure of $P_{0}$), and $h_{i}$ and $s_{i}$ are the enthalpy and entropy, respectively, of stream i at temperature *T* and pressure *P*.

In addition to the exergy destruction of equipment *i*, in the cycle, the contribution of exergy destruction, $E_{cont,i}$, in the total exergy loss of the cycle, ${\dot{E}}_{{}_{d,tot}}$ can be determined based on Equation (11).

(11) $$E_{cont,i}=\frac{{\dot{E}}_{{}_{d,i}}}{{\dot{E}}_{{}_{d,tot}}}=\frac{{\dot{E}}_{{}_{d,i}}}{\sum {\dot{E}}_{{}_{d,i}}}$$

In this manner, for each equipment of the investigated cycle, the exergy analysis is applied according to Equations (9) and (10) [31,32,33].

For the turbine, because it was considered to follow an isentropic process, there is no heat transfer. Then, Equations (9) and (10) are simplified to Equation (12) for the exergy destruction by the turbine, ${\dot{E}}_{{}_{d,turb}}$,

(12) $${\dot{E}}_{{}_{d,turb}}={\dot{m}}_{{}_{r}}(h_{1}-h_{2})-{\dot{m}}_{{}_{r}}T_{0}(s_{1}-s_{2})-\dot{W_{s}}$$

For the condenser, the exergy destruction, ${\dot{E}}_{{}_{d,C}}$, is derived by,

(13) $${\dot{E}}_{{}_{d,C}}={\dot{m}}_{{}_{r}}(h_{2}-h_{3})-{\dot{m}}_{{}_{r}}T_{0}(s_{2}-s_{3})-{\dot{Q}}_{C}(1-\frac{T_{0}}{T_{LS}})$$

where $T_{LS}$ is the heat sink temperature which absorbs ${\dot{Q}}_{C}$, and is considered as 298.15 K.

For the evaporator, the exergy destruction, ${\dot{E}}_{{}_{d,e}}$ is calculated by Equation (14) (during the day).

(14) $${\dot{E}}_{{}_{d,e}}={\dot{m}}_{{}_{r}}\left[\left(h_{4}-h_{1})-T_{0}(s_{4}-s_{1}\right)\right]+{\dot{m}}_{{}_{w}}\left[\left(h_{6}-h_{5})-T_{0}(s_{6}-s_{5}\right)\right]$$

For the PCM tank, it is important to consider the assumption of insulation of the tank. Therefore, the exergy destructions of the PCM tank are determined based on Equations (15) and (16) for day and night, respectively.

(15) $$E_{d,PCM,day}={\dot{m}}_{{}_{w}}((h_{10}-h_{7})-T_{0}(s_{10}-s_{7}))$$

(16) $$E_{d,PCM,night}={\dot{m}}_{{}_{r}}((h_{4^{\prime }}-h_{1^{\prime }})-T_{0}(s_{4^{\prime }}-s_{1^{\prime }}))$$

In Equations (15) and (16), $E_{d,PCM,day}$ and $E_{d,PCM,night}$ are the exergy destructions of the PCM tank during the day and night, respectively. As a result, for a 24-h period, the exergy destruction of the PCM, $E_{d,PCM}$, can be calculated based on Equation (17) [34,35].

(17) $$E_{d,PCM}=E_{d,PCM,day}+E_{d,PCM,night}$$

Finally, according to Equations (9) and (10), the exergy destruction of the water tank, ${\dot{E}}_{{}_{d,wt}}$, is calculated based on Equation (18).

(18) $${\dot{E}}_{{}_{d,wt}}={\dot{m}}_{w_{9}}(h_{8}-h_{9})-{\dot{m}}_{w_{9}}T_{0}(s_{8}-s_{9})+{\dot{Q}}_{S}(1-\frac{T_{0}}{T_{HW}})$$

where $T_{HW}$ is the heat source temperature and equal to 0.75$T_{sun}$ [29]. Moreover, for calculating the enthalpy and entropy changes of liquid water at constant pressure in the water tank, Equations (19) and (20) are used.

(19) $$\Delta h=\int_{T_{8}}^{T_{9}}C_{pw}dT$$

(20) $$\Delta s=\int_{T_{8}}^{T_{9}}\frac{C_{pw}}{T}dT$$

In these equations, $C_{pw}$ is the heat capacity of water, and $T_{8}$ and $T_{9}$ are the inlet and outlet temperatures of the water streams of the water tank.

After determining the exergy destruction of all of the equipment, the total exergy destruction of the cycle, which includes the non-idealities of the system, can be determined according to Equation (21).

(21) $${\dot{E}}_{{}_{d,tot}}=\sum {\dot{E}}_{{}_{d,i}}$$

According to this equation, the total exergy destruction of the cycle is actually the sum of the exergy destruction of each equipment in the cycle.

### 2.4. Investigated DESs

In this study, seven DESs, as well as paraffin, were considered as PCMs to study a solar thermal power generation cycle. The information of the studied DESs, including the HBA and HBD components, and their molar ratios and molecular weights are presented in Table 1.

## 3. Results and Discussion

The first step for performing the calculations in the presented modified cycle, is determining the physical properties of the DESs. The enthalpy of fusion and melting point are required for each DES. Table 2 presents the values of enthalpies of fusion for the HBA and HBD components, as well as the melting points of the investigated DESs. In order to calculate the enthalpies of fusion of the DESs, a simple thermodynamic mixing rule was used for the HBA and HBD components, as given by Equation (22) [38].

(22) $$\Delta h_{fus,PCM}=y_{HBA}\Delta h_{fus,HBA}+y_{HBD}\Delta h_{fus,HBD}$$

where $y_{HBA}$ and $y_{HBD}$ are the mole fractions of the HBA and HBD components, respectively, and $\Delta h_{fus,HBA}$ and $\Delta h_{fus,HBD}$ are their corresponding enthalpies of fusion, respectively. The calculated values of enthalpies of fusion for the investigated DESs are also reported in Table 2.

In addition to the studied DESs, paraffin, with a carbon number range of 21 to 50 and a melting point of 68 °C, with an enthalpy of fusion equal to 189 J/g, was considered as a conventional PCM [42].

All of the required properties of R134a (the working fluid of Rankine cycle) and water (the working fluid of the heating cycle), including enthalpies, entropies, vapor pressures at different temperatures and pressures, and heat capacities were obtained from the NIST database [40].

In order to have a fair investigation of all the DESs, the operational conditions of the studied cycles for each DES were considered the same. Table 3 reports the operational conditions of the investigated cycles.

According to the presented operational conditions, the outlet water-temperature from the water tank, $T_{9}$, for all the investigated cycles was assumed to be higher than the melting-point temperature of the investigated DESs (PCMs), to ascertain the transfer of heat from hot water to the DES. Moreover, the outlet temperature of R134a from the PCM tank, $T_{1^{\prime }}$ was considered to be lower than the PCM melting point temperature, in order to be sure of heat transfer from the PCM to R134a. Moreover, the condenser temperature, the evaporator pressure, and the mass flow rates of water and R134a were selected according to the thermodynamic properties of the working fluid and the selected PCMs, as well as taking into account the values given in previously published studies [15,33].

After obtaining all of the required information for the investigated cycles, the performances of the cycles using the investigated DESs as PCMs were investigated by energy and exergy analyses.

The most important equipment in the investigated cycles, which are flexible in changing the operational conditions, are the condenser and evaporator. Therefore, by changing the condenser temperature and evaporator pressure (according to Table 3), the performances of the investigated cycles were studied, with a focus on the produced power, the required mass of DES, and the total exergy loss of the cycle.

### 3.1. Method of Calculation

To calculate the cycle’s characteristics, such as power production, required mass of PCM, and exergy losses, the following calculation steps were followed:

Step 1. Based on the selected condenser temperature, evaporator pressure, and the provided assumptions, the enthalpies and entropies of Streams 1 (1′), 3, and 4 (4′) were determined;

Step 2. The entropy and enthalpy of Stream 2 were calculated based on the turbine’s isentropic efficiency, which was considered as 0.75 in this work;

Step 3. Using the calculated enthalpies, the produced power and the required mass of PCM were calculated based on Equations (1) and (5);

Step 4. According to the given exergy analysis method, the exergy losses were determined.

### 3.2. Effect of the Condenser Temperature

**Figure 2:**
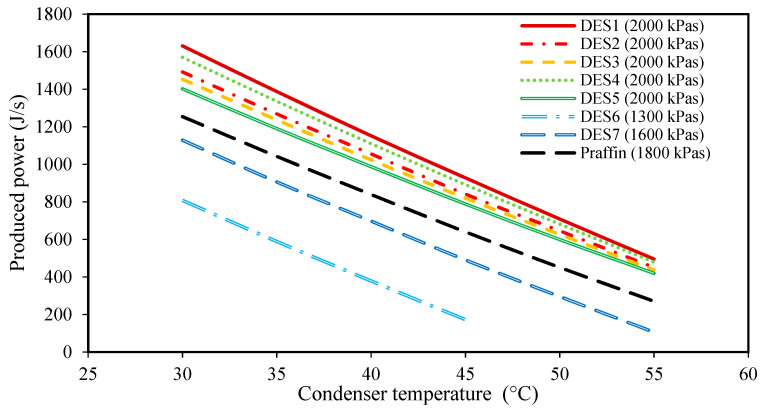
The effect of condenser temperature on the produced power. (The evaporator pressure for each system is shown in the legend for each PCM).

**Figure 3:**
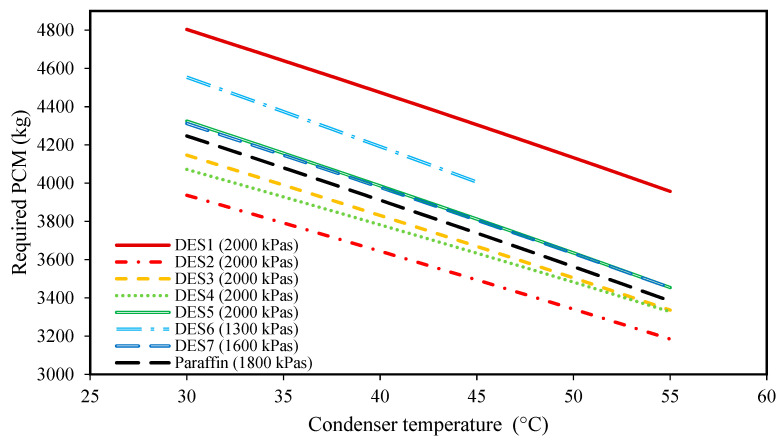
The effect of condenser temperature on the required amount of DES. (The evaporator pressure of each system is shown in the legend of each PCM).

**Figure 4:**
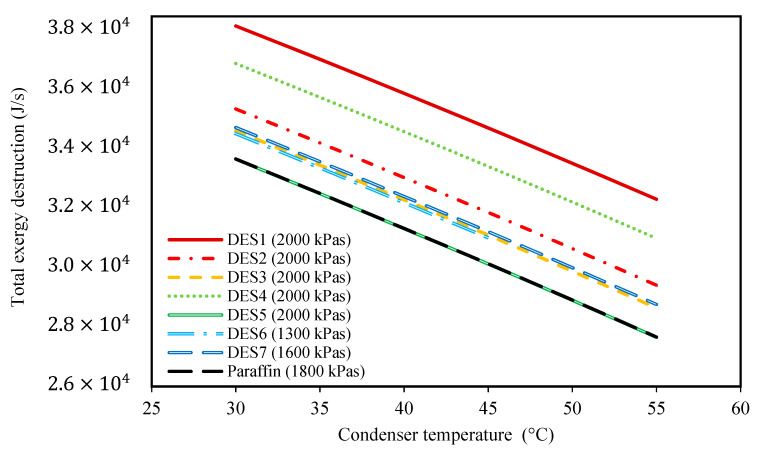
The effect of condenser temperature on the total exergy destruction. (The evaporator pressure of each system is shown in the legend of each PCM).

**Figure 5:**
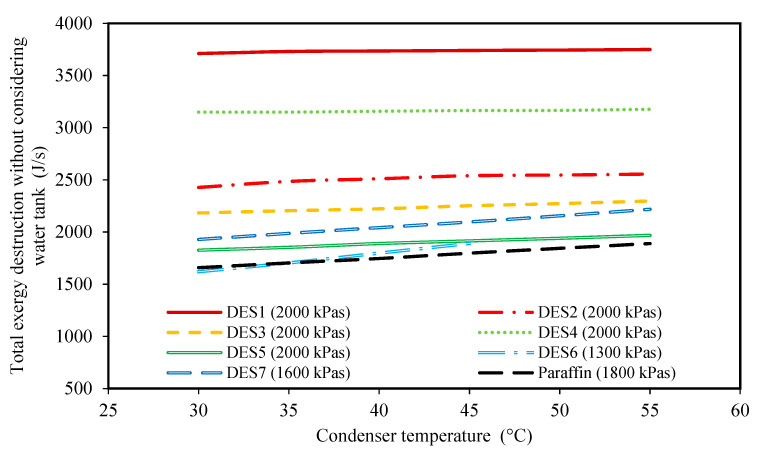
The effect of condenser temperature on the total exergy destruction without considering the water tank exergy loss. (The evaporator pressure of each system is shown in the legend of each PCM).

The effects of changing the condenser temperature on the produced power, the required mass of PCM, and the total exergy loss of each cycle are shown in Figure 2, Figure 3, Figure 4 and Figure 5 for all of the studied cycles. However, it is important to keep in mind that the inlet R134a to the turbine should be at a superheated vapor state, therefore, the evaporator pressures of each cycle will be different. The values of the evaporator pressure in each cycle are also shown in Figure 2, Figure 3, Figure 4 and Figure 5.

Based on the achieved results, it can be seen that by increasing the condenser temperature, the produced power and the required mass of PCM both decrease. In fact, by increasing the condenser temperature while all the other operational conditions of the cycle are constant, the enthalpy of Stream 1 (or 1’), which is a function of the evaporator pressure and temperature, $T_{1}$(or $T_{1^{\prime }}$), remains constant for each cycle. Moreover, increasing the condenser temperature increases the condenser pressure as well. Accordingly, Stream 2 leaves the turbine at a higher pressure and temperature. Therefore, the enthalpy of Stream 2 will increase when the condenser temperature is increased. Based on Equation (1), for a constant mass flow rate of the working fluid, $\dot{m_{r}}$, and a constant enthalpy, $h_{1}$(or $h_{1^{\prime }}$), the produced power decreases by increasing $h_{2}$. This can be seen in all of the studied cycles in Figure 2. By comparing the different DESs investigated, it is shown that except for DES6 and DES7, the other DESs produce higher amounts of power than the conventional paraffin PCM at the same operational conditions.

Additionally, as discussed earlier, increasing the condenser temperature does not have any effect on the properties of Streams 1 and 1’. However, increasing the condenser temperature increases $T_{3}$, and then, $T_{4^{\prime }}$ as well, which means that Stream 4 reaches higher enthalpy values. Accordingly, based on Equation (5), a lower amount of PCM is required at higher condenser temperatures, which is also evidenced by Figure 3.

Another important finding from this figure is the lower required amounts of DES4, DES3, and DES2 as the PCMs with respect to paraffin. The other investigated cycles require greater amounts of DES than paraffin. In fact, one of the most important properties that play a vital role in the performance of a cycle is the enthalpy of fusion of the PCM. By comparing the enthalpies of fusion of the studied DESs, it is seen that DES2, DES3, and DES4, have the highest enthalpies of fusion among the PCMs. Accordingly, in the cycles with either DES2, DES3, or DES4 as the PCM, a lower mass of PCM is required to provide a desired amount of power, in comparison to other cycles.

Additionally, Figure 4, demonstrates the effect of condenser temperature on the total exergy destruction of the investigated cycles. Based on the results of this figure, by increasing the condenser temperature, the total exergy destruction of each of the studied cycles decreases. Indeed, by increasing the condenser temperature, the produced power and the required amount of PCM both decrease. Accordingly, the required amount of input heat to the water tank decreases as well. Based on Equation (18), by increasing the condenser temperature, the exergy destruction of the water tank also decreases. By comparing the investigated cycles, it is shown that only the cycle of DES5 has a similar total exergy destruction to the paraffin cycle.

Moreover, it is common practice to study the total exergy destruction of only the Rankine cycle instead of the whole cycle. For this purpose, Figure 5 is presented. This figure demonstrates the effect of condenser temperature on the total exergy destruction of the cycle without considering the exergy loss of the water tank. Based on the achieved results, at higher condenser temperatures, the total exergy destruction is higher.

In fact, when the difference between the condenser and the surrounding temperatures increases, the process of discarding heat $\dot{Q_{c}}$ to the surrounding moves further away from a “reversible” process. Accordingly, the total exergy destruction increases at higher condenser temperatures. By comparing the results of Figure 4 and Figure 5, it can be seen that the effect of condenser temperature on the total exergy destruction of the whole cycle is the exact opposite of the results of Figure 4. In fact, it can be concluded that the exergy loss of the water tank is much greater than the other parts of the cycle, and, thus, controls the behavior of total exergy destruction of the cycle. Therefore, as discussed earlier, when the condenser temperature increases, lower amounts of heat are necessary for increasing the enthalpy of Stream 4, so the temperature change of water in the evaporator decreases, leading to lower exergy destruction of the water tank, which has the highest effect on the total exergy losses.

In general, based on the achieved results of Figure 2, Figure 3, Figure 4 and Figure 5, it can be concluded that lower condenser temperatures of the investigated cycles are more favorable from the point of view of produced power. However, the condenser temperature cannot be lower than a specific value. In fact, to ensure that the discarding of heat, $\dot{Q_{C}}$, to the surrounding does indeed occur, the condenser temperature should not be lower than the surrounding temperature. However, it should be noted that decreasing the evaporator temperature leads to higher exergy destructions, and also, larger amounts of required DES. Therefore, based on these findings, the temperature of 30 °C can be suggested as a suitable condenser temperature for all of the studied cycles to achieve high power production.

### 3.3. Effect of the Evaporator Pressure

**Figure 6:**
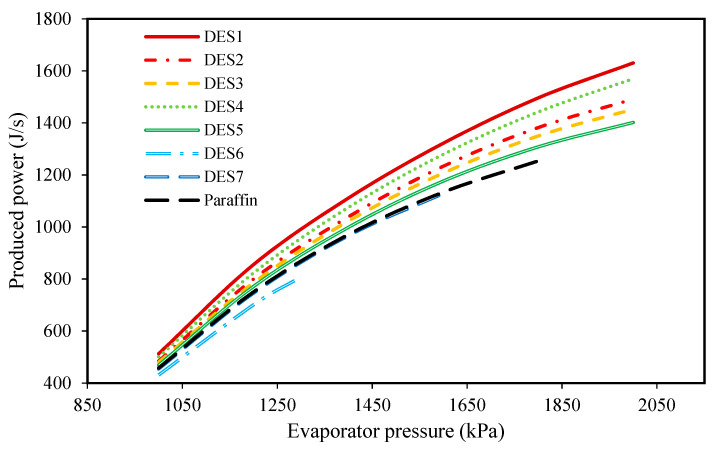
The effect of evaporator pressure on the produced power.

**Figure 7:**
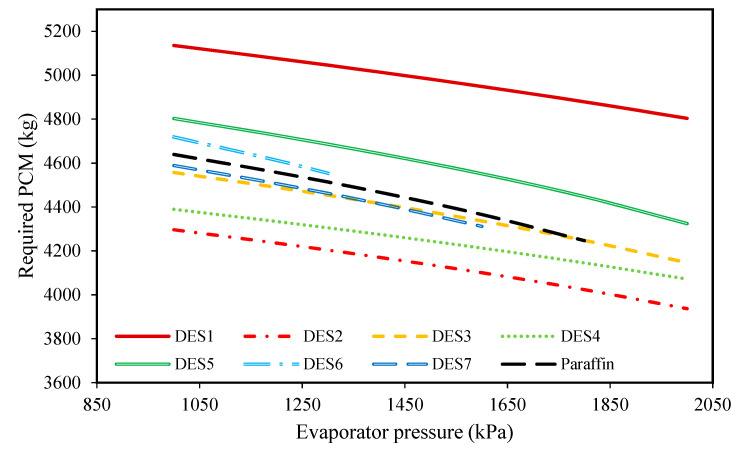
The effect of evaporator pressure on the required amount of DES.

**Figure 8:**
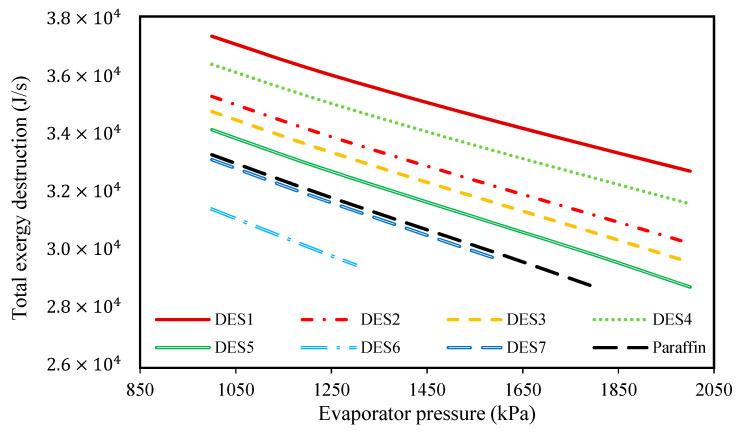
The effect of evaporator pressure on the total exergy destructions of the investigated cycles.

**Figure 9:**
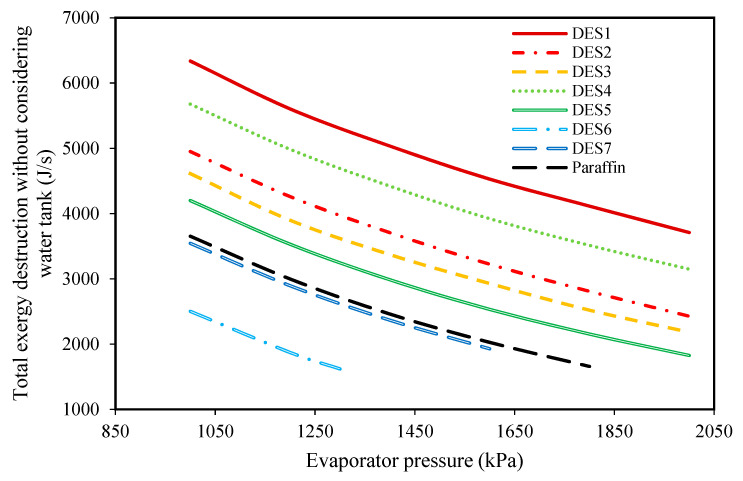
The effect of evaporator pressure on the total exergy loss of the cycle without considering the exergy destructions of the water tank.

To study the effect of evaporator pressure (according to Table 3) on the performances of the investigated cycles, the produced power, the required amount of PCM, and the total exergy destruction upon evaporator pressure changes were studied and the results are presented in Figure 6, Figure 7, Figure 8 and Figure 9, respectively.

These investigations were carried out at a condenser temperature of 30 °C, which was proposed above as a possible optimum condenser temperature.

Based on Figure 6, by increasing the evaporator pressure, the produced power increases for all of the studied cycles. Indeed, increasing the evaporator pressure does not have any effect on the pressure of Stream 2, while it does increase the pressure of Stream 1 during the day. Therefore, the inlet pressure of the turbine increases while the outlet pressure remains constant, so the produced power increases while considering a constant working fluid mass flow rate. Additionally, since we assumed that the cycle’s operational conditions are the same during night and day, the same scenario can be assumed for the pressures of Streams 1 and 2 during the night, which leads to the production of more power during the night as well. Moreover, it can be seen that all of the investigated DESs, except for DES6 and DES7, produce greater, or at least the same amount of power as paraffin. The reason that DES6 and DES7 produce lower power in comparison to the other DESs and the studied paraffin, is their smaller enthalpies of fusion.

Indeed, as mentioned earlier, the enthalpy of fusion of a PCM is an important factor whose value affects the behavior of the cycle. In fact, by the increased enthalpy of fusion of a PCM, a higher amount of energy can be stored within a fixed period of time. Therefore, a PCM with a high enthalpy of fusion can provide greater energy to the refrigerant of the Rankine cycle. Subsequently, and based on the performance of the Rankine cycle, a greater amount of power can be achieved when a larger amount of energy is added to its refrigerant.

Additionally, according to Figure 7, it can be seen that by increasing the evaporator pressure, the required mass of PCM decreases for all of the investigated cycles. Because the pressures of Streams 1 and 1’ are the same during day and night, increasing the evaporator pressure at a constant evaporator temperature leads to reduced enthalpies of Streams 1 and 1’. Accordingly, based on Equation (6), for a constant mass flow rate of the working fluid, smaller amounts of the PCM are required. Additionally, based on the results of Figure 7, it can be seen that except for DES1, DES5, and DES6, the required amount of PCM for the investigated cycle is either lower or the same as the cycle which uses paraffin, due to the differences between the enthalpies of fusion.

In addition to the required PCM and the produced power, the effect of changing of evaporator pressure on the total exergy destructions of the investigated cycles was studied and shown in Figure 8.

According to the results, increasing the evaporator pressure decreases the total exergy destruction of all the studied cycles. Additionally, in Figure 9, the effect of changing evaporator pressure on the total exergy destruction of the investigated cycles, without considering the exergy destruction of the water tank, is presented. Based on the results, increasing the evaporator pressure leads to decreases in the total exergy destruction (without the water tank) for all of the studied cycles. Actually, it was shown that by increasing the evaporator pressure, the required mass of the PCMs consequently decreases, which means that the working fluid (R134a) requires lower amounts of heat for vaporization. In other words, since it was assumed that the investigated cycle’s operational conditions are the same during night and day, by increasing the evaporator pressure, the required amount of heat which is required for vaporizing R134a decreases during the day. Actually, in the daytime, water is responsible for providing the required amount of heat for the evaporation of R134a, and by increasing the evaporator pressure, the temperature-change of water decreases. From a thermodynamics point of view, by decreasing the water temperature, the evaporator tends toward a reversible process, so its exergy destruction decrease.

Based on the achieved results, it can be concluded that increasing the evaporator pressure is favorable for the cycle’s performance and the highest possible evaporator pressure should be chosen, however, since the inlet fluid to the turbine should be super-heated vapor, there is a limit on evaporator pressure increase. Additionally, evaporator pressure is restricted by safety protocols and operational limitations.

In Figure 9, the effect of the melting point temperature of the PCM is shown on the cycle performance. DES6, DES7, and paraffin have a lower melting-point temperatures in comparison to the other studied PCMs, and since the temperature of the Rankine cycle’s refrigerant is equal to *T_m,PCM_* − 5, the outlet refrigerant from the evaporator cannot be superheated vapor at high evaporator pressures when a PCM with a low melting-point is used. Based on this limitation, it is suggested to consider the evaporator pressure as 2000 kPa. By comparing the results of the investigated cycles in Table 4, it can be seen that the cycle which uses DES2 (1 Choline chloride: 0.9 urea) as its PCM requires the lowest amount of DES. Additionally, the cycle which uses DES5 (1 Choline chloride:0.8 oxalic acid) as its PCM has the lowest total exergy destruction.

**Figure 10:**
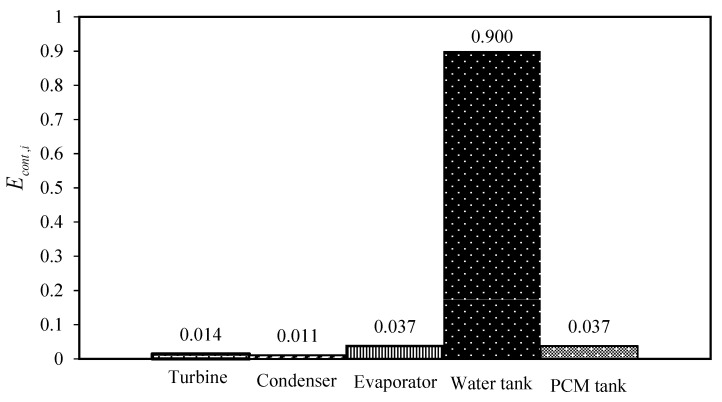
The contribution of each unit of the cycle in the exergy destruction.

However, the cycle which uses DES1 (1 Choline chloride:1 suberic acid) as the PCM has the highest produced power. Nevertheless, according to the results of Table 4, choosing DES4 (1 Choline chloride:0.5 4-hydroxybenzoic acid) as the PCM is the most rational because following DES1, it has the highest power production while the required mass of DES is much lower than DES1. Furthermore, from the exergy destruction point of view, its total exergy destruction is in the same order as the other cycles. For a detailed examination, the contribution of all of the equipment of the cycle using DES4 as the PCM is shown in Figure 10. Moreover, the exergy destruction contribution of the other investigated cycles is also given in Figures S1–S6 of the Supplementary Materials.

Based on Figure 10, it is obvious that the water tank has the highest contribution in comparison to the other equipment. One of the most important sources of such high exergy destruction in the water tank is the temperature of the heat source of the water tank. Accordingly, for improving the performance of the cycle, the operation of the water tank should be optimized.

### 3.4. Effects of the Melting Point Temperature and the Enthalpy of Fusion

In the previous sections, the performances of the investigated DESs were compared to one another at various operational conditions. In this section, we discuss the effects of the melting point temperature and the enthalpy of fusion of a DES on the cycle performance. According to the achieved results, a DES with a higher melting point temperature and higher enthalpy of fusion, such as DES1, DES3, and DES4, is more favorable and leads to a better cycle performance. A higher melting point temperature of a DES leads to a higher enthalpy of the fluid entering the turbine. However, melting point temperature is not the only criteria for selecting a suitable DES. In this work, it was shown that DES4 can potentially be the best DES among the investigated DESs according to performance, however, its melting-point temperature is lower than that of DES1. Actually, the heat of fusion of a DES is also an important factor that should be considered for selecting the best DES. In fact, increasing the enthalpy of fusion of a DES leads to lower required amounts of DES for the same amount of power generation. In general, when choosing an appropriate DES for power generation in the given cycle, the melting-point temperature and enthalpy of fusion of the DES should be high enough, while some other operational conditions, such as viscosity, should be considered as well.

## 4. Conclusions

In this work, a modified cycle was introduced for a solar thermal power plant that uses a PCM tank for storing solar energy during the day and releases the energy during the night. Based on the modified cycle, power generation based on solar energy can occur continuously not only during the day, but also, throughout the night. Additionally, in order to investigate the feasibility of replacing conventional PCMs with green and sustainable materials, various DESs were considered as novel PCMs for use in solar thermal power plants. The feasibility study was carried out by applying exergy and energy analyses to the modified cycles. For this purpose, seven different DESs were suggested as potential PCMs, to be compared with paraffin as a conventional PCM. Based on the considered PCMs, the optimum operating conditions of the modified solar thermal power plant cycles were investigated by studying the effects of changing the condenser temperature and evaporator pressure on the produced power, the required amount of DES, and the total exergy destruction of the cycles. Based on the achieved results, it was suggested that the highest of cycle performances can potentially be achieved at a condenser temperature of 30 °C and an evaporator pressure of 2000 kPa. At these suggested operational conditions, the cycle which uses DES4 (Choline chloride:4-hydroxybenzoic acid 1:0.5) as its PCM shows the best performance. By comparing the achieved results, it was found that some of the selected DESs have better performance than paraffin from the points of view of energy and exergy analyses. Due to the larger enthalpy of fusion of DES4 in comparison to paraffin, the cycle which operates with DES4 produces 25% more power in comparison to the cycle which uses paraffin as the PCM, together with a lower required amount of DES (175 kg lower), and their total exergy losses are in the same order.

Additionally, by comparing the contributions of each equipment of the solar thermal power plant cycle in the aspect of total exergy destruction, it was concluded that the water tank which absorbs the solar energy, has the highest contribution to the total exergy destruction of the cycle.

Based on the results of this work, it can be concluded that DES4 has the potential to be used as a PCM in solar power plants due to its suitable performance in comparison to paraffin, in addition to its environmental benefits.

## Figures and Tables

**Table 1:** The HBA, HBD, and molar ratios of the investigated DESs in this study.

DES Code	HBA	HBD	HBA:HBD Molar Ratio	DES Molecular Weight (g/mol)
DES1	Choline chloride	Suberic acid	1:1 ^1^	156.92
DES2	Choline chloride	Urea	1:0.9 ^2^	102.22
DES3	Choline chloride	Gallic acid	1:0.5 ^1^	149.79
DES4	Choline chloride	4-Hydroxybenzoic acid	1:0.5 ^1^	139.13
DES5	Choline chloride	Oxalic acid	1:0.8 ^2^	117.81
DES6	Choline chloride	Itaconic acid	1:1 ^1^	134.87
DES7	Choline chloride	p-Coumaric acid	1:0.5 ^1^	147.81

^1^ Reference [36] ^2^ Reference [37].

**Table 2:** Enthalpies of fusion and melting points of the investigated DESs in this study.

DES	HBA to HBD molar ratio	$\Delta h_{fus,HBA}(\frac{kJ}{mol})$	$\Delta h_{fus,HBD}(\frac{kJ}{mol})$	$\Delta h_{fus,DES}(\frac{kJ}{mol})$	$\Delta h_{fus,DES}(\frac{J}{g})$	$T_{m,DES}({}^{{}^{\circ}}C)$
DES1	1:1	29.76 ^1^	30.70 ^2^	30.23	192.65	93 ^4^
DES2	1:0.9	29.76 ^1^	13.61 ^2^	22.17	216.89	80 ^5^
DES3	1:0.5	29.76 ^1^	30.96 ^3^	30.17	201.42	77 ^4^
DES4	1:0.5	29.76 ^1^	32.00 ^2^	30.50	219.22	87 ^4^
DES5	1:0.8	29.76 ^1^	12.31 ^3^	22.08	187.42	73 ^5^
DES6	1:1	29.76 ^1^	17.49 ^3^	23.62	175.13	57 ^4^
DES7	1:0.5	29.76 ^1^	24.78 ^3^	28.10	190.11	67 ^4^
Paraffin	-	-	-	-	189.00 ^6^	68 ^6^

^1^ Reference [39]; ^2^ Reference [40]; ^3^ Calculated using the Joback–Reid method [41]; ^4^ Reference [36]; ^5^ Reference [37]; ^6^ Reference [42].

**Table 3:** The operational conditions for the investigated cycles.

Water Tank Outlet Temperature, *T*_9_	Condenser Temperature Range (°C)	R134a Outlet Temperature of PCM tank, *T*_1′_	Evaporator Pressure (kPa)	Mass Flow Rate of Water, $\dot{m_{w}}(kg/s)$	Mass Flow Rate of R134a $\dot{m_{r}}(kg/s)$
*T_m,PCM_* + 5	30–55	*T_m,PCM_* − 5	1000–2000	1.5	0.1

**Table 4:** The results of exergy and energy analyses for all of the investigated cycles at the condenser temperature of 30 °C and their evaporator pressure.

Cycle	Evaporator Pressure (kPa)	Produced Power (J/s)	Required Mass of PCM (kg)	Total Exergy Destruction (J/s)	Total Exergy Destruction Without the Water Tank (J/s)
DES1	2000	1630.5	4803.57	32,717.11	3710.68
DES2	2000	1491.75	3936.70	30,249.98	2427.12
DES3	2000	1452.75	4146.14	29,600.00	2182.53
DES4	2000	1569.75	4071.55	31,599.43	3148.88
DES5	2000	1402.01	4324.36	28,758.28	1826.06
DES6	1300	807.75	4553.63	29,515.55	1619.44
DES7	1600	1128.75	4311.56	29,697.07	1928.57
Paraffin	1800	1254.14	4246.63	28,763.60	1658.43

## Data Availability

Not applicable.

## Supplementary Materials

The following supporting information can be downloaded online: Figure S1 to Figure S7.
