# Supplementary material for: Deep Eutectic Solvents as Phase Change Materials in Solar Thermal Power Plants: Energy and Exergy Analyses
Source: Molecules. 2022 Feb 20;27(4):1427. doi: 10.3390/molecules27041427 (PMC8880683; doi:10.3390/molecules27041427)
Supplement: Supplementary file 1 [file molecules-27-01427-s001.zip › molecules-1595587-supplementary.pdf]

Supplementary Information

Deep eutectic solvents as phase change materials in solar thermal power plants: Energy and Exergy analyses

Hamed Peyrovedin[1], Reza Haghbakhsh[2,3], Ana Rita C. Duarte[3] and Alireza Shariati[1,*]

[1] School of Chemical and Petroleum Engineering, Shiraz University, Shiraz 71345-51154, Iran
[2] Department of Chemical Engineering, Faculty of Engineering, University of Isfahan, 81746-73441, Isfahan, Iran
[3] LAQV, REQUIMTE, Departamento de Química da Faculdade de Ciências e Tecnologia, Universidade Nova de Lisboa, 2829-516 Caparica, Portugal
* Correspondence: shariati@shirazu.ac.ir; Tel.: +98 71 36133704

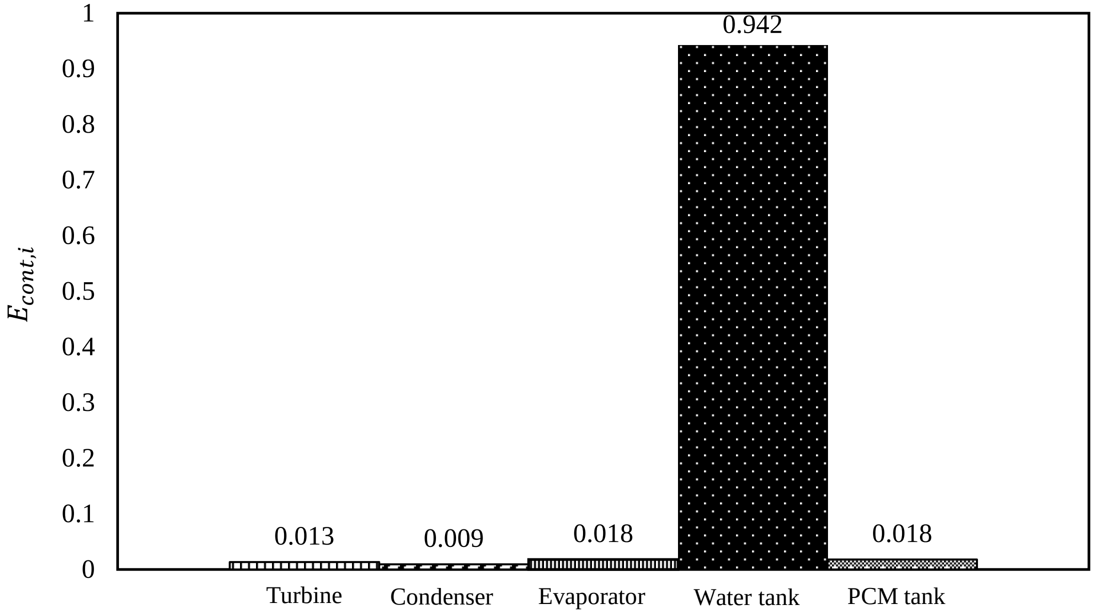

Figure S1. The contribution of each part of the cycle in the exergy destruction of the system which uses paraffin as the PCM, at a condenser pressure of 1800 kPa and condenser temperature of 30 $^{\circ}C$.

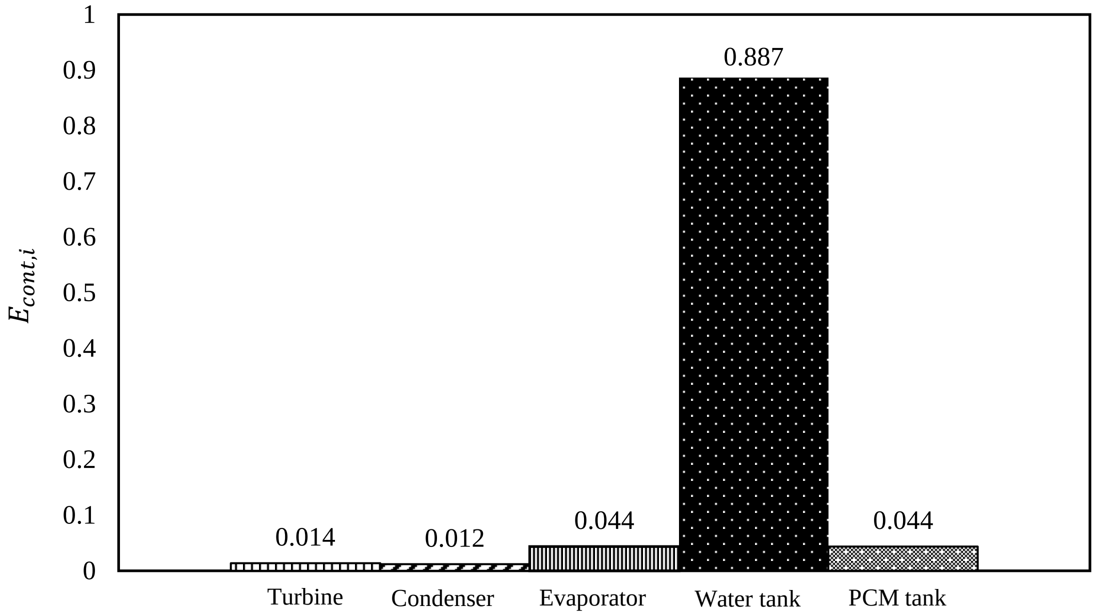

Figure S2. The contribution of each part of the cycle in the exergy destruction of the system which uses DES1 as the PCM at a condenser pressure of 2000 kPa and condenser temperature of 30 $^{\circ}C$.

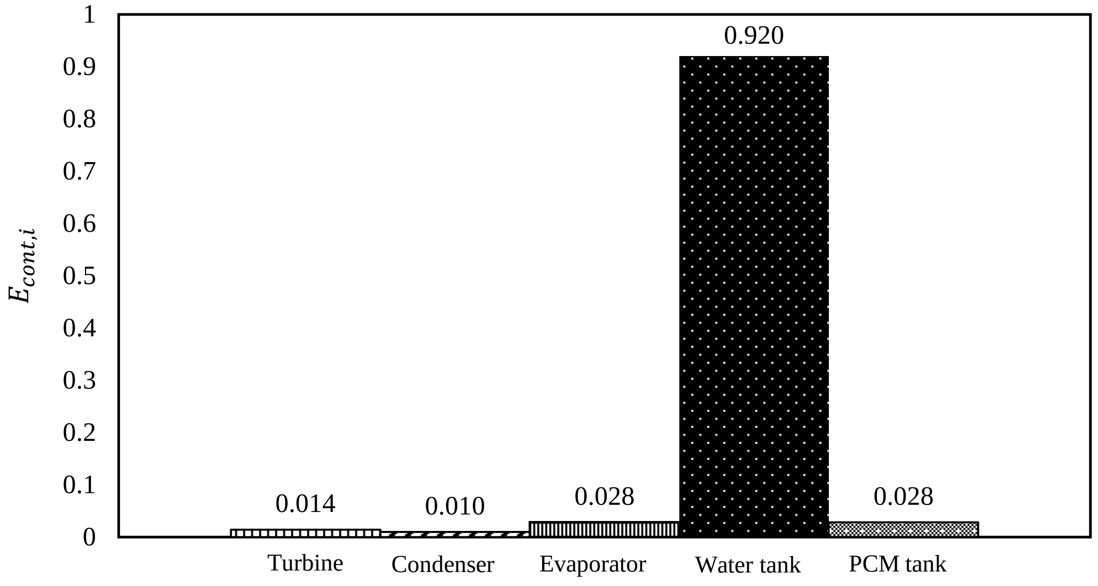

Figure S3. The contribution of each part of the cycle in the exergy destruction of the system which uses DES2 as the PCM at a condenser pressure of 2000 kPa and condenser temperature of 30 $^{\circ}C$.

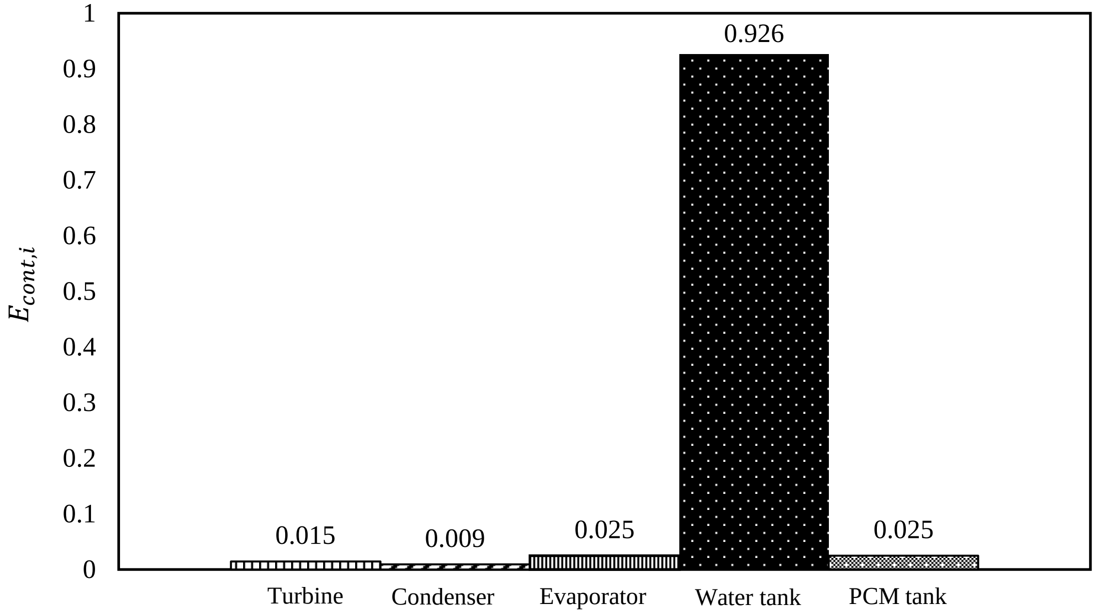

Figure S4. The contribution of each part of the cycle in the exergy destruction of the system which uses DES3 as the PCM at a condenser pressure of 2000 kPa and condenser temperature of 30 $^{\circ}C$.

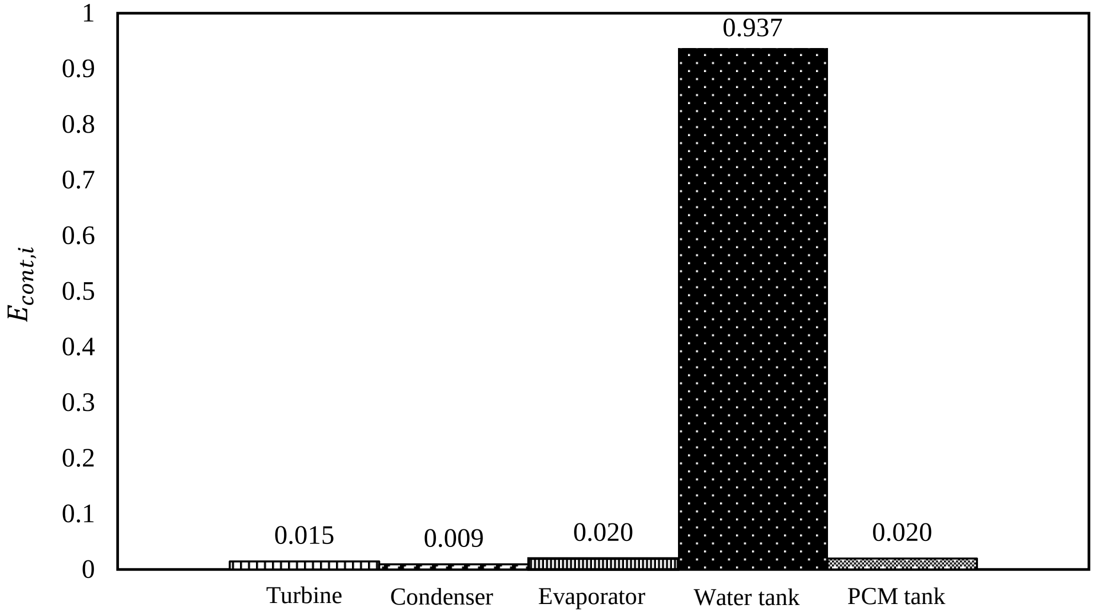

Figure S5. The contribution of each part of the cycle in the exergy destruction of the system which uses DES5 as the PCM at a condenser pressure of 2000 kPa and condenser temperature of 30 $^{\circ}C$.

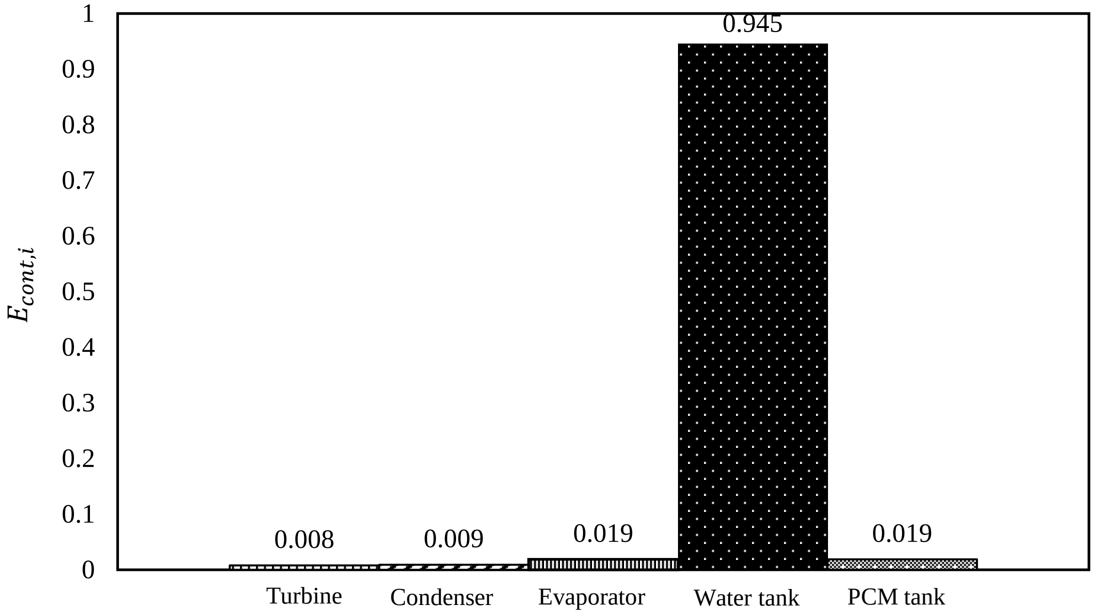

Figure S6. The contribution of each part of the cycle in the exergy destruction of the system which uses DES6 as the PCM at a condenser pressure of 1300 kPa and condenser temperature of 30 $^{\circ}C$.

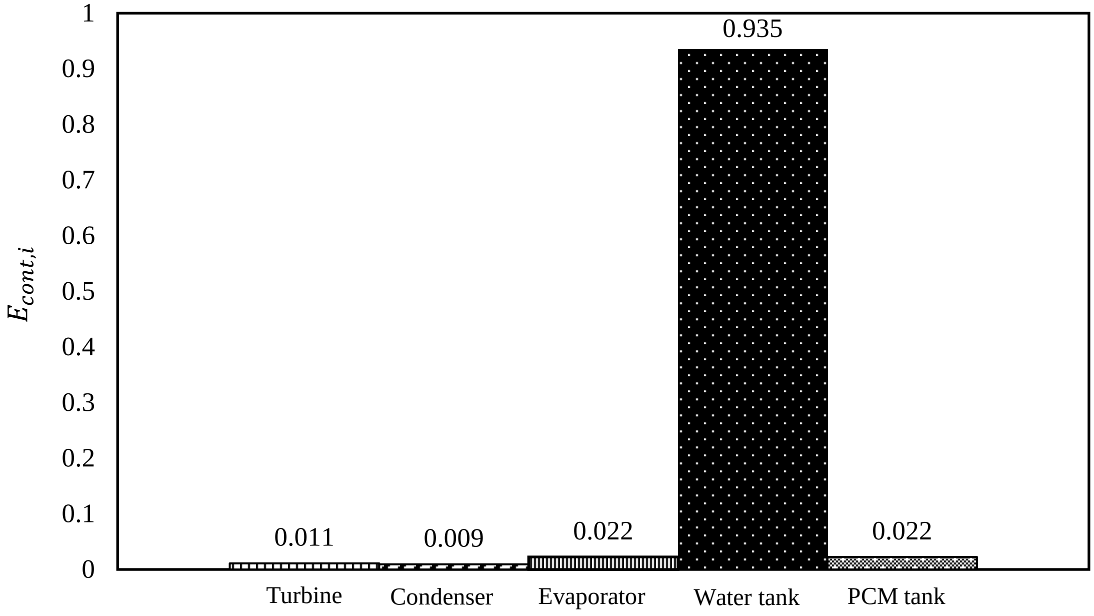

Figure S7. The contribution of each part of the cycle in the exergy destruction of the system which uses DES7 as the PCM at a condenser pressure of 1600 kPa and condenser temperature of 30 $^{\circ}C$.
